# Correction: Greiner et al. Consecutive-Day Ventricular and Atrial Cardiomyocyte Isolations from the Same Heart: Shifting the Cost–Benefit Balance of Cardiac Primary Cell Research. *Cells* 2022, *11*, 233

**DOI:** 10.3390/cells13121034

**Published:** 2024-06-14

**Authors:** Joachim Greiner, Teresa Schiatti, Wenzel Kaltenbacher, Marica Dente, Alina Semenjakin, Thomas Kok, Dominik J. Fiegle, Thomas Seidel, Ursula Ravens, Peter Kohl, Rémi Peyronnet, Eva A. Rog-Zielinska

**Affiliations:** 1 Institute for Experimental Cardiovascular Medicine, University Heart Center Freiburg—Bad Krozingen and Faculty of Medicine, Albert-Ludwig University of Freiburg, 79110 Freiburg im Breisgau, Germany; joachim.greiner@uniklinik-freiburg.de (J.G.); teresa.schiatti@uniklinik-freiburg.de (T.S.); david.kaltenbacher@uniklinik-freiburg.de (W.K.); alina07@gmx.de (A.S.); thomas.kok@uniklinik-freiburg.de (T.K.); ursula.ravens@uniklinik-freiburg.de (U.R.); peter.kohl@uniklinik-freiburg.de (P.K.); 2 Department of Experimental and Clinical Medicine, Division of Physiology, University of Florence, 50134 Florence, Italy; marica.dente@unifi.it; 3 Institute of Cellular and Molecular Physiology, Friedrich-Alexander-University of Erlangen-Nürnberg, 91054 Erlangen, Germanythomas.seidel@fau.de (T.S.); 4 CIBSS Centre for Integrative Biological Signalling Studies, University of Freiburg, 79110 Freiburg im Breisgau, Germany

In the original publication [1], there was an error in the Materials and Methods Section, Sub-Section 2.3. Cell Isolation. The concentration of the digestion solution was wrong. The correct concentration appears below. The authors state that the scientific conclusions are unaffected. This correction was approved by the Academic Editor. The original publication has also been updated.

Tissue was then rinsed twice in wash solution 1 (each 2 mL), and further digested with Liberase TL Research Grade (concentration: 0.23 mg/mL in 2.2 mL wash solution 1 supplemented with 5 µM CaCl_2_; Hoffmann-La Roche, Basel, Switzerland) for up to 45 min (ventricles)/15 min (atria).
