# Supplementary material for: Consecutive-Day Ventricular and Atrial Cardiomyocyte Isolations from the Same Heart: Shifting the Cost–Benefit Balance of Cardiac Primary Cell Research
Source: Cells. 2022 Jan 11;11(2):233. doi: 10.3390/cells11020233 (PMC8773758; doi:10.3390/cells11020233)
Supplement: Supplementary file 1 [file cells-11-00233-s001.zip › cells-1477310-supplementary.pdf]

Table S1. Composition of solutions.

Concentration					
		**Modified Tyrode**	**Wash solution 1**	**Wash solution 2**	**Modified recording Tyrode**
NaCl	mM	138	100	100	137
KCl	mM	5.4	15	15	4
$MgCl_2$	mM	2	2	2	1
HEPES	mM	10	10	10	10
Glucose	mM	10	10	10	10
BDM	mM	30	30	30	-
KH_2PO_4	mM	-	2.5	2.5	
L-Glutamic acid monopotassium salt	mM	-	20	20	-
Taurine	mM	-	20	20	20
Adenosine	mM	-	-	-	5
Creatine	mM	-	-	-	10
L-carnitine	mM	-	-	-	2
NaH_2PO_4	mM	0.33	-	-	-
Bovine serum albumin	mg/mL	-	2	10	-
$CaCl_2$	mM	0.5	-	0.005	1.8

Figure S1.

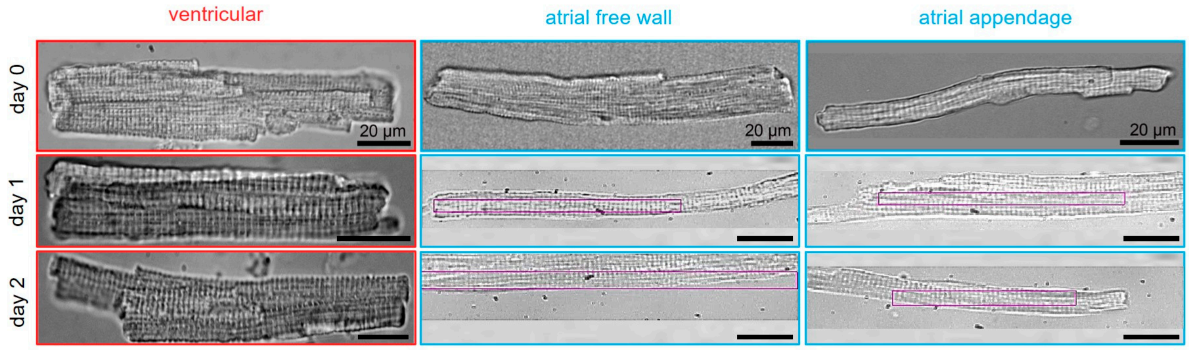

Figure S1. Representative brightfield and phase-contrast microscopy images of living ventricular and atrial CM after isolation at day 0, 1 and 2. All scale bars correspond to 20 µm, atrial cells at day 1 and 2 are phase-contrast microscopy images. Regions of interest used to record SL are shown in magenta.
